# Crystal structure of 10a-hy­droxy-9-(3-nitro­phen­yl)-3,6-diphenyl-3,4,5,6,7,8a,9,10a-octa­hydro-1*H*-xanthene-1,8(2*H*)-dione

**DOI:** 10.1107/S2056989015021246

**Published:** 2015-11-21

**Authors:** Xin-Yuan Zhang, Bing-Xiang Hu, Ze-Yu Zhou, Lei Zhou, Fang-Ming Wang

**Affiliations:** a Jiangsu University of Science and Technology, Zhenjiang 212003, People’s Republic of China

**Keywords:** crystal structure, xanthene, hydrogen bonding

## Abstract

In the octa­hydroxanthenedione unit of the title compound, C_31_H_27_NO_6_, the central di­hydro­pyran ring shows an envelope conformation, while the bilateral cyclo­hexene and cyclo­hexane rings adopt a half-boat conformation and a chair conformation, respectively. The nitro­benzene ring is twisted with respect to the two benzene rings, making dihedral angles of 63.1 (1) and 63.0 (1)°. In the crystal, O—H⋯O hydrogen bonds link the mol­ecules into supra­molecular chains propagating along the *a*-axis direction.

## Abstract 2

The central di­hydro­pyran ring of the compound shows an envelope conformation. In the crystal, O—H⋯O hydrogen bonds link the mol­ecules into supra­molecular chains propagating along the *a*-axis direction.

## Chemical context   

Xanthenes are important biologically active heterocyclic compounds, which possess anti-inflammatory, anti­bacterial and anti­viral activities (Shakibaei *et al.*, 2007 ▸; Lambert *et al.*, 1997 ▸). Many studies have been carried out on xanthene derivatives (Knight & Little, 2001 ▸; Jha & Beal, 2004 ▸; Lu *et al.*, 2011 ▸; Cui *et al.*, 2012 ▸; Wang *et al.*, 2015 ▸). Herein, we report the synthesis and the crystal structure of the title xanthene deriv­ative.
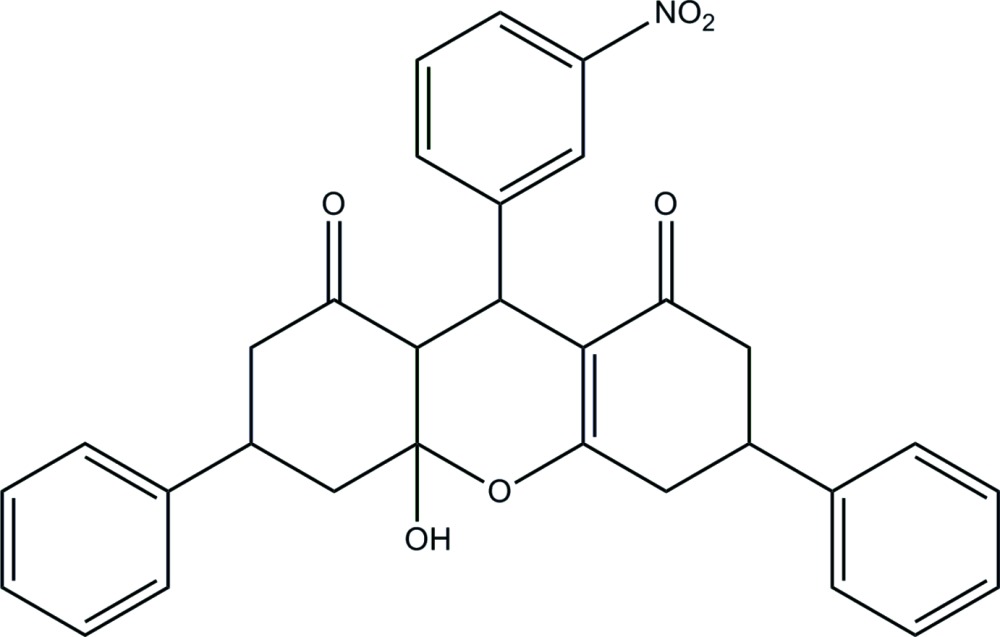


## Structural commentary   

**Figure 1:**
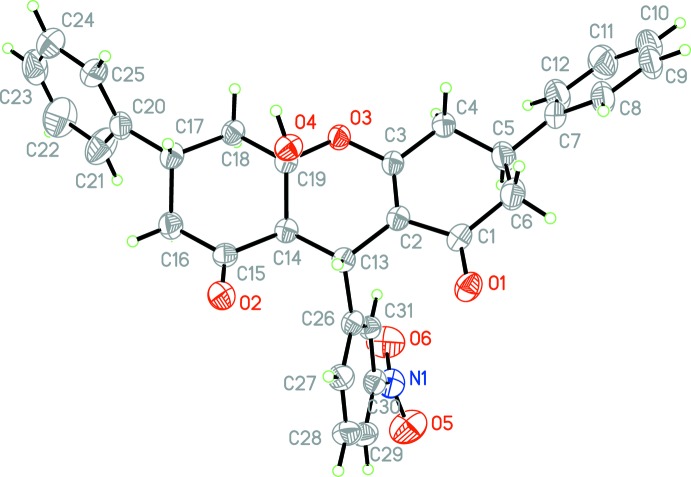
The mol­ecular structure of the title compound, showing the atom labelling, with displacement ellipsoids drawn at the 50% probability level.

The mol­ecular structure of the title compound is shown in Fig. 1 ▸. The C1—O1 and C15—O2 bond lengths are 1.234 (4) and 1.202 (4) Å, respectively. The central di­hydro­pyran ring shows an envelope conformation with atom C19 as the flap, while the bilateral cyclo­hexene and cyclo­hexane rings adopt a half boat conformation and a chair conformation, respectively. The nitro­benzene ring is twisted with respect to the C7–C10 and C20–C25 benzene rings, making dihedral angles of 63.1 (1) and 63.0 (1)°, respectively.

## Supra­molecular features   

**Figure 2:**
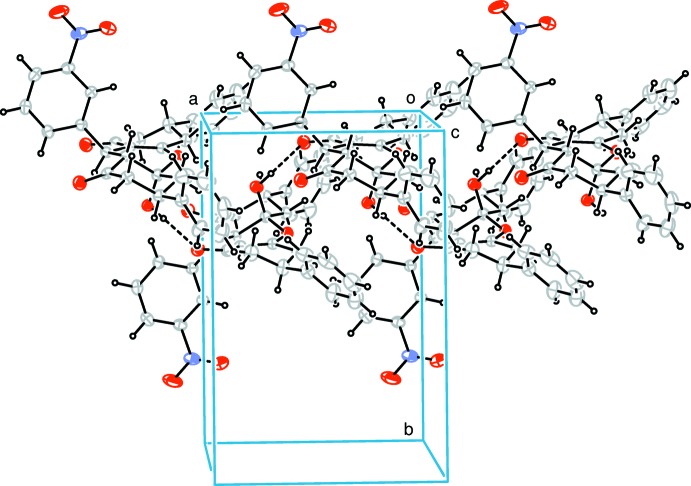
Packing diagram showing the hydrogen bonds as dashed lines.

In the crystal (Fig. 2 ▸), the mol­ecules are linked by O—H⋯O hydrogen bonds (Table 1 ▸), generating supra­molecular chains propagating along the *a*-axis direction.

## Database survey   

A search of the Cambridge Structural Database for 10a-hy­droxy-3,4,5,6,7,8a,9,10a-octa­hydro-1*H*-xanthene-1,8(2*H*)-dione gave 16 hits. None of them are substituted at the 3,6-position with two phenyl groups. Several compounds substituted at the 9-position with an aryl group are similar to the title compound, for example, 9-(2,6-di­chloro­phen­yl)-4a-hydroxy-3,3,6,6-tetra­methyl-1,2,3,4,4a,5,6,7,8,9a-deca­hydroxanthene-1,8-dione (Bolte *et al.*, 2001 ▸), 9-(2,3-di­chloro­phen­yl)-4a-hy­droxy-3,3,6,6-tetra­methyl-3,4,4a,6,7,9,9a,10-octa­hydroanthracene-1,8(2*H*,5*H*)-dione (Mohammadi Ziarani *et al.*, 2008 ▸) and 9-(2-chloro­phen­yl)-4a-hy­droxy-3,4,4a,5,6,7,9,9a-octa­hydro-1*H*-xanthracene-1,8(2*H*)-dione (Liu *et al.*, 2014 ▸).

## Synthesis and crystallization   

The title compound was synthesized in accordance to our previous procedure (Wang *et al.*, 2015 ▸). 5-Phenyl­cyclo­hexane-1,3-dione (7.52 g, 40 mmol) and 3-nitro­benzaldehyde (20 mmol) were dissolved in the mixture of methanol (10 ml) and ethanol (10 ml) in the presence of trace l-proline (5 mmol) and stirred for 4 h. After completion of the reaction, the white solid products were filtered under reduced pressure and washed with ethanol (78% yield). m.p. 445.15–447.15 K. IR (KBr pellets, cm^−1^): 3370 (O—H), 1648 (C=O), 1562 (C=C). MS (ESI) *m*/*z*: 510.2 [*M* + H^+^]. ^1^H NMR (DMSO-*d*_6_, 400 MHz): δ 2.56–2.92 (*m*, 8H, 2a-H, 7a-H, 2b-H, 7b-H, 4a-H, 5a-H, 4b-H, 5b-H); 3.43 (*m*, 2H, 6-H, 3-H); 5.46 (*m*, 1H, 9-H); 7.14 (*m*, 2H, 11-H, 10-OH); 7.22–8.02 (*m*, 14H, PhH). Analysis calculated for C_31_H_27_NO_6_: C 73.07, H 5.34, N 2.75%; found: C 72.92, H 5.30, N 2.65%. Single crystals of the title compound were obtained by slow evaporation from an ethanol solution at room temperature in the form of colorless blocks.

## Refinement details   

Crystal data, data collection and structure refinement details are summarized in Table 2 ▸. All H atoms of were fixed geometrically and treated as riding with C—H = 0.97 (methyl­ene), 0.98 (methine), 0.93 (phen­yl) and O—H = 0.82 Å, with *U*_iso_(H) = 1.2*U*_eq_(C,O).

## Supplementary Material

Crystal structure: contains datablock(s) I, New_Global_Publ_Block. DOI: 10.1107/S2056989015021246/xu5879sup1.cif

Structure factors: contains datablock(s) I. DOI: 10.1107/S2056989015021246/xu5879Isup2.hkl

Supporting information file. DOI: 10.1107/S2056989015021246/xu5879Isup3.cml

CCDC reference: 1435921

Additional supporting information:  crystallographic information; 3D view; checkCIF report

## Figures and Tables

**Table 1:** Hydrogen-bond geometry (Å, °)

*D*—H⋯*A*	*D*—H	H⋯*A*	*D*⋯*A*	*D*—H⋯*A*
O4—H4*C*⋯O1^i^	0.82	1.89	2.714 (3)	180

Symmetry code: (i) 
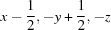
.

**Table 2:** Experimental details

Crystal data	
Chemical formula	C_31_H_27_NO_6_
*M*_r_	509.53
Crystal system, space group	Orthorhombic, *P*2_1_2_1_2_1_
Temperature (K)	291
*a*, *b*, *c* (Å)	8.973 (4), 13.520 (6), 21.251 (9)
*V* (Å^3^)	2578 (2)
*Z*	4
Radiation type	Mo *K*α
μ (mm^−1^)	0.09
Crystal size (mm)	0.25 × 0.20 × 0.16

Data collection	
Diffractometer	Bruker SMART APEXII area detector
Absorption correction	Multi-scan (*SADABS*; Bruker, 2000 ▸)
*T*_min_, *T*_max_	0.979, 0.986
No. of measured, independent and observed [*I* > 2σ(*I*)] reflections	19805, 5040, 3978
*R*_int_	0.028
(sin θ/λ)_max_ (Å^−1^)	0.617

Refinement	
*R*[*F* ^2^ > 2σ(*F* ^2^)], *wR*(*F* ^2^), *S*	0.044, 0.125, 1.03
No. of reflections	5040
No. of parameters	344
H-atom treatment	H-atom parameters constrained
Δρ_max_, Δρ_min_ (e Å^−3^)	0.43, −0.21

Computer programs: *APEX2* and *SAINT* (Bruker, 2000 ▸), *SHELXS2014* (Sheldrick, 2008 ▸) and *SHELXL2014* (Sheldrick, 2015 ▸).

## supplementary crystallographic information

### Crystal data

**Table:**

C_31_H_27_NO_6_	*D*_x_ = 1.313 Mg m^−^^3^
*M**_r_* = 509.53	Mo *K*α radiation, λ = 0.71073 Å
Orthorhombic, *P*2_1_2_1_2_1_	Cell parameters from 7999 reflections
*a* = 8.973 (4) Å	θ = 2.5–25.5°
*b* = 13.520 (6) Å	µ = 0.09 mm^−^^1^
*c* = 21.251 (9) Å	*T* = 291 K
*V* = 2578 (2) Å^3^	Block, colorless
*Z* = 4	0.25 × 0.20 × 0.16 mm
*F*(000) = 1072	

### Data collection

**Table:**

Bruker SMART APEXII area-detector diffractometer	5040 independent reflections
Radiation source: sealed tube	3978 reflections with *I* > 2σ(*I*)
Graphite monochromator	*R*_int_ = 0.028
φ and ω scans	θ_max_ = 26.0°, θ_min_ = 2.4°
Absorption correction: multi-scan (*SADABS*; Bruker, 2000)	*h* = −10→11
*T*_min_ = 0.979, *T*_max_ = 0.986	*k* = −16→16
19805 measured reflections	*l* = −26→26

### Refinement

**Table:**

Refinement on *F*^2^	Hydrogen site location: inferred from neighbouring sites
Least-squares matrix: full	H-atom parameters constrained
*R*[*F*^2^ > 2σ(*F*^2^)] = 0.044	*w* = 1/[σ^2^(*F*_o_^2^) + (0.0674*P*)^2^ + 0.3283*P*] where *P* = (*F*_o_^2^ + 2*F*_c_^2^)/3
*wR*(*F*^2^) = 0.125	(Δ/σ)_max_ = 0.010
*S* = 1.03	Δρ_max_ = 0.43 e Å^−^^3^
5040 reflections	Δρ_min_ = −0.21 e Å^−^^3^
344 parameters	Absolute structure: Flack *x* determined using 1503 quotients [(*I*^+^)-(*I*^-^)]/[(*I*^+^)+(*I*^-^)] (Parsons *et al.*, 2013)
0 restraints	Absolute structure parameter: 1.3 (4)

### Special details

**Table:**

Experimental. Least-squares planes (x,y,z in crystal coordinates) and deviations from them (* indicates atom used to define plane) 6.7298 (0.0116) x + 5.2391 (0.0295) y + 11.3904 (0.0347) z = 4.9647 (0.0106) * -0.0122 (0.0031) C20 * 0.0030 (0.0039) C21 * 0.0057 (0.0040) C22 * -0.0052 (0.0037) C23 * -0.0042 (0.0035) C24 * 0.0128 (0.0031) C25 Rms deviation of fitted atoms = 0.0081 5.3891 (0.0148) x - 9.6057 (0.0212) y - 7.7932 (0.0389) z = 1.2144 (0.0092) Angle to previous plane (with approximate esd) = 88.773 ( 14.5 ) * 0.0060 (0.0029) C7 * -0.0023 (0.0029) C8 * -0.0024 (0.0031) C9 * 0.0034 (0.0036) C10 * 0.0004 (0.0038) C11 * -0.0052 (0.0034) C12 Rms deviation of fitted atoms = 0.0038 - 1.0457 (0.0125) x + 0.3544 (0.0170) y + 21.0986 (0.0100) z = 0.3439 (0.0082) Angle to previous plane (with approximate esd) = 63.082 ( 14.2 ) * 0.0003 (0.0022) C26 * -0.0038 (0.0023) C27 * 0.0040 (0.0025) C28 * -0.0005 (0.0025) C29 * -0.0030 (0.0022) C30 * 0.0030 (0.0020) C31 Rms deviation of fitted atoms = 0.0028
Geometry. All e.s.d.'s (except the e.s.d. in the dihedral angle between two l.s. planes) are estimated using the full covariance matrix. The cell e.s.d.'s are taken into account individually in the estimation of e.s.d.'s in distances, angles and torsion angles; correlations between e.s.d.'s in cell parameters are only used when they are defined by crystal symmetry. An approximate (isotropic) treatment of cell e.s.d.'s is used for estimating e.s.d.'s involving l.s. planes.

### Fractional atomic coordinates and isotropic or equivalent isotropic displacement parameters (Å^2^)

**Table:**

	*x*	*y*	*z*	*U*_iso_*/*U*_eq_	
C1	0.3896 (4)	0.0908 (2)	−0.07887 (14)	0.0532 (7)	
C2	0.3277 (3)	0.09893 (19)	−0.01590 (13)	0.0444 (6)	
C3	0.1786 (3)	0.1037 (2)	−0.00994 (13)	0.0448 (6)	
C4	0.0696 (3)	0.0842 (2)	−0.06132 (15)	0.0537 (7)	
H4A	0.0206	0.1457	−0.0725	0.064*	
H4B	−0.0061	0.0390	−0.0460	0.064*	
C5	0.1411 (4)	0.0405 (3)	−0.11952 (16)	0.0699 (10)	
H5A	0.1651	−0.0280	−0.1084	0.084*	
C6	0.2850 (4)	0.0872 (3)	−0.13382 (17)	0.0749 (10)	
H6A	0.2672	0.1541	−0.1484	0.090*	
H6B	0.3323	0.0511	−0.1678	0.090*	
C7	0.0334 (4)	0.0332 (3)	−0.17443 (15)	0.0657 (9)	
C8	0.0491 (5)	0.0871 (3)	−0.22896 (16)	0.0795 (12)	
H8A	0.1254	0.1333	−0.2324	0.095*	
C9	−0.0483 (8)	0.0729 (4)	−0.27876 (19)	0.1001 (17)	
H9A	−0.0365	0.1097	−0.3154	0.120*	
C10	−0.1603 (7)	0.0061 (5)	−0.2747 (2)	0.109 (2)	
H10A	−0.2248	−0.0033	−0.3084	0.131*	
C11	−0.1778 (6)	−0.0470 (5)	−0.2208 (3)	0.1080 (17)	
H11A	−0.2546	−0.0929	−0.2176	0.130*	
C12	−0.0816 (5)	−0.0329 (4)	−0.1710 (2)	0.0873 (13)	
H12A	−0.0952	−0.0692	−0.1342	0.105*	
C13	0.4348 (3)	0.1112 (2)	0.03895 (13)	0.0448 (6)	
H13A	0.4897	0.1731	0.0329	0.054*	
C14	0.3463 (3)	0.1195 (2)	0.10028 (13)	0.0454 (6)	
H14A	0.3183	0.0525	0.1133	0.054*	
C15	0.4323 (4)	0.1669 (2)	0.15441 (14)	0.0502 (7)	
C16	0.3445 (4)	0.1767 (3)	0.21452 (15)	0.0594 (8)	
H16A	0.4040	0.2117	0.2455	0.071*	
H16B	0.3230	0.1114	0.2310	0.071*	
C17	0.1982 (4)	0.2325 (2)	0.20407 (14)	0.0531 (7)	
H17A	0.2239	0.2996	0.1905	0.064*	
C18	0.1095 (4)	0.1841 (2)	0.15049 (13)	0.0500 (7)	
H18A	0.0795	0.1180	0.1629	0.060*	
H18B	0.0202	0.2223	0.1422	0.060*	
C19	0.2036 (3)	0.1785 (2)	0.09110 (13)	0.0477 (7)	
C20	0.1030 (4)	0.2418 (3)	0.26275 (14)	0.0583 (8)	
C21	0.0884 (7)	0.1681 (4)	0.3066 (2)	0.0987 (15)	
H21A	0.1430	0.1101	0.3018	0.118*	
C22	−0.0045 (7)	0.1777 (5)	0.3573 (2)	0.1152 (18)	
H22A	−0.0120	0.1263	0.3862	0.138*	
C23	−0.0851 (5)	0.2603 (6)	0.3660 (2)	0.1031 (18)	
H23A	−0.1489	0.2661	0.4003	0.124*	
C24	−0.0718 (6)	0.3356 (5)	0.3235 (2)	0.1023 (17)	
H24A	−0.1272	0.3931	0.3289	0.123*	
C25	0.0237 (5)	0.3272 (3)	0.27247 (18)	0.0790 (11)	
H25A	0.0341	0.3798	0.2446	0.095*	
C26	0.5470 (3)	0.0276 (2)	0.04296 (13)	0.0469 (7)	
C27	0.6988 (3)	0.0467 (3)	0.04997 (15)	0.0556 (8)	
H27A	0.7324	0.1117	0.0502	0.067*	
C28	0.7995 (4)	−0.0294 (3)	0.05661 (17)	0.0660 (9)	
H28A	0.9002	−0.0150	0.0617	0.079*	
C29	0.7535 (4)	−0.1262 (3)	0.05574 (17)	0.0654 (9)	
H29A	0.8214	−0.1777	0.0599	0.079*	
C30	0.6046 (4)	−0.1444 (2)	0.04855 (14)	0.0538 (7)	
C31	0.4998 (3)	−0.0703 (2)	0.04240 (14)	0.0484 (7)	
H31A	0.3993	−0.0856	0.0379	0.058*	
N1	0.5518 (4)	−0.2479 (2)	0.04813 (15)	0.0718 (8)	
O1	0.5249 (2)	0.09640 (17)	−0.08882 (11)	0.0611 (6)	
O2	0.5583 (3)	0.19545 (18)	0.14823 (11)	0.0635 (6)	
O3	0.1096 (2)	0.12872 (16)	0.04468 (9)	0.0519 (5)	
O4	0.2463 (2)	0.27061 (15)	0.06846 (10)	0.0537 (5)	
H4C	0.1792	0.3106	0.0746	0.081*	
O5	0.6420 (4)	−0.3133 (2)	0.03810 (18)	0.1123 (12)	
O6	0.4215 (4)	−0.2637 (2)	0.0582 (2)	0.1056 (11)	

### Atomic displacement parameters (Å^2^)

**Table:**

	*U*^11^	*U*^22^	*U*^33^	*U*^12^	*U*^13^	*U*^23^
C1	0.0563 (18)	0.0513 (17)	0.0522 (17)	−0.0012 (15)	0.0112 (15)	−0.0060 (14)
C2	0.0519 (17)	0.0372 (14)	0.0441 (14)	0.0000 (12)	0.0036 (13)	0.0003 (12)
C3	0.0515 (16)	0.0427 (15)	0.0402 (14)	−0.0003 (12)	0.0023 (12)	−0.0001 (12)
C4	0.0528 (17)	0.0608 (18)	0.0476 (16)	0.0010 (15)	−0.0005 (13)	−0.0023 (14)
C5	0.064 (2)	0.098 (3)	0.0475 (18)	−0.0060 (19)	−0.0007 (16)	0.0034 (18)
C6	0.075 (2)	0.098 (3)	0.0523 (19)	−0.001 (2)	0.0060 (17)	−0.0111 (19)
C7	0.072 (2)	0.085 (2)	0.0401 (16)	0.014 (2)	0.0003 (16)	−0.0048 (17)
C8	0.116 (3)	0.076 (2)	0.0467 (19)	0.015 (2)	−0.009 (2)	−0.0058 (17)
C9	0.152 (5)	0.103 (3)	0.046 (2)	0.049 (4)	−0.020 (3)	−0.011 (2)
C10	0.122 (4)	0.138 (5)	0.067 (3)	0.039 (4)	−0.037 (3)	−0.037 (3)
C11	0.087 (3)	0.148 (5)	0.088 (3)	−0.010 (3)	−0.019 (3)	−0.028 (3)
C12	0.076 (3)	0.126 (4)	0.060 (2)	−0.011 (3)	−0.008 (2)	0.000 (2)
C13	0.0469 (15)	0.0406 (14)	0.0468 (15)	−0.0042 (12)	0.0022 (12)	−0.0013 (12)
C14	0.0508 (16)	0.0393 (14)	0.0461 (15)	−0.0014 (13)	0.0015 (12)	0.0004 (12)
C15	0.0555 (19)	0.0452 (16)	0.0499 (16)	0.0099 (15)	−0.0032 (14)	0.0003 (13)
C16	0.068 (2)	0.0623 (19)	0.0475 (17)	0.0058 (17)	−0.0025 (15)	−0.0006 (15)
C17	0.0623 (19)	0.0505 (16)	0.0464 (16)	0.0015 (15)	0.0059 (14)	−0.0012 (13)
C18	0.0537 (16)	0.0511 (16)	0.0451 (15)	0.0001 (14)	0.0066 (14)	0.0009 (13)
C19	0.0478 (15)	0.0505 (16)	0.0448 (15)	0.0019 (13)	0.0008 (12)	−0.0022 (13)
C20	0.0622 (19)	0.068 (2)	0.0450 (16)	−0.0021 (18)	−0.0014 (15)	−0.0082 (15)
C21	0.136 (4)	0.082 (3)	0.078 (3)	0.008 (3)	0.043 (3)	0.011 (2)
C22	0.132 (5)	0.132 (5)	0.082 (3)	−0.014 (4)	0.038 (3)	0.015 (3)
C23	0.068 (3)	0.183 (6)	0.058 (3)	−0.013 (3)	0.014 (2)	−0.030 (3)
C24	0.085 (3)	0.144 (5)	0.078 (3)	0.033 (3)	0.006 (3)	−0.032 (3)
C25	0.081 (3)	0.096 (3)	0.060 (2)	0.025 (2)	0.0047 (19)	−0.009 (2)
C26	0.0509 (16)	0.0467 (15)	0.0430 (14)	−0.0013 (13)	0.0070 (13)	0.0006 (12)
C27	0.0506 (17)	0.0625 (19)	0.0538 (18)	−0.0055 (15)	0.0042 (14)	0.0030 (15)
C28	0.0467 (17)	0.083 (2)	0.068 (2)	0.0033 (17)	0.0045 (16)	0.0026 (18)
C29	0.063 (2)	0.069 (2)	0.064 (2)	0.0190 (18)	0.0052 (17)	0.0072 (16)
C30	0.067 (2)	0.0470 (16)	0.0469 (15)	0.0042 (14)	0.0035 (15)	0.0039 (13)
C31	0.0508 (16)	0.0501 (16)	0.0444 (14)	0.0012 (13)	0.0013 (13)	0.0014 (12)
N1	0.095 (2)	0.0502 (17)	0.0700 (18)	0.0082 (18)	0.0015 (18)	0.0109 (14)
O1	0.0579 (13)	0.0663 (14)	0.0591 (13)	−0.0065 (11)	0.0139 (10)	−0.0047 (10)
O2	0.0595 (14)	0.0683 (15)	0.0627 (14)	−0.0025 (12)	−0.0050 (11)	−0.0077 (11)
O3	0.0455 (10)	0.0657 (13)	0.0444 (10)	−0.0024 (10)	0.0029 (9)	−0.0066 (9)
O4	0.0587 (12)	0.0471 (11)	0.0554 (12)	0.0045 (10)	0.0076 (10)	0.0075 (9)
O5	0.139 (3)	0.0557 (16)	0.142 (3)	0.0285 (18)	0.020 (2)	0.0107 (18)
O6	0.097 (2)	0.0608 (16)	0.159 (3)	−0.0136 (16)	−0.007 (2)	0.0041 (18)

### Geometric parameters (Å, º)

**Table:**

C1—O1	1.234 (4)	C16—H16A	0.9700
C1—C2	1.453 (4)	C16—H16B	0.9700
C1—C6	1.499 (5)	C17—C20	1.517 (4)
C2—C3	1.345 (4)	C17—C18	1.536 (4)
C2—C13	1.520 (4)	C17—H17A	0.9800
C3—O3	1.358 (3)	C18—C19	1.520 (4)
C3—C4	1.489 (4)	C18—H18A	0.9700
C4—C5	1.513 (5)	C18—H18B	0.9700
C4—H4A	0.9700	C19—O4	1.389 (4)
C4—H4B	0.9700	C19—O3	1.462 (4)
C5—C6	1.468 (5)	C20—C21	1.371 (5)
C5—C7	1.519 (5)	C20—C25	1.371 (5)
C5—H5A	0.9800	C21—C22	1.369 (7)
C6—H6A	0.9700	C21—H21A	0.9300
C6—H6B	0.9700	C22—C23	1.343 (8)
C7—C12	1.367 (6)	C22—H22A	0.9300
C7—C8	1.376 (5)	C23—C24	1.365 (9)
C8—C9	1.385 (6)	C23—H23A	0.9300
C8—H8A	0.9300	C24—C25	1.387 (6)
C9—C10	1.354 (8)	C24—H24A	0.9300
C9—H9A	0.9300	C25—H25A	0.9300
C10—C11	1.360 (8)	C26—C31	1.390 (4)
C10—H10A	0.9300	C26—C27	1.394 (4)
C11—C12	1.380 (6)	C27—C28	1.377 (5)
C11—H11A	0.9300	C27—H27A	0.9300
C12—H12A	0.9300	C28—C29	1.373 (6)
C13—C26	1.515 (4)	C28—H28A	0.9300
C13—C14	1.530 (4)	C29—C30	1.366 (5)
C13—H13A	0.9800	C29—H29A	0.9300
C14—C19	1.521 (4)	C30—C31	1.380 (4)
C14—C15	1.527 (4)	C30—N1	1.477 (5)
C14—H14A	0.9800	C31—H31A	0.9300
C15—O2	1.202 (4)	N1—O6	1.207 (4)
C15—C16	1.507 (5)	N1—O5	1.218 (4)
C16—C17	1.530 (5)	O4—H4C	0.8200
			
O1—C1—C2	121.9 (3)	C15—C16—H16B	109.3
O1—C1—C6	119.0 (3)	C17—C16—H16B	109.3
C2—C1—C6	118.7 (3)	H16A—C16—H16B	108.0
C3—C2—C1	118.1 (3)	C20—C17—C16	113.9 (3)
C3—C2—C13	123.5 (3)	C20—C17—C18	110.7 (3)
C1—C2—C13	118.2 (3)	C16—C17—C18	110.0 (3)
C2—C3—O3	123.1 (3)	C20—C17—H17A	107.3
C2—C3—C4	125.1 (3)	C16—C17—H17A	107.3
O3—C3—C4	111.8 (2)	C18—C17—H17A	107.3
C3—C4—C5	112.9 (3)	C19—C18—C17	110.4 (2)
C3—C4—H4A	109.0	C19—C18—H18A	109.6
C5—C4—H4A	109.0	C17—C18—H18A	109.6
C3—C4—H4B	109.0	C19—C18—H18B	109.6
C5—C4—H4B	109.0	C17—C18—H18B	109.6
H4A—C4—H4B	107.8	H18A—C18—H18B	108.1
C6—C5—C4	112.0 (3)	O4—C19—O3	109.8 (2)
C6—C5—C7	115.4 (3)	O4—C19—C18	113.4 (2)
C4—C5—C7	112.5 (3)	O3—C19—C18	105.2 (2)
C6—C5—H5A	105.3	O4—C19—C14	106.4 (2)
C4—C5—H5A	105.3	O3—C19—C14	109.3 (2)
C7—C5—H5A	105.3	C18—C19—C14	112.8 (2)
C5—C6—C1	113.8 (3)	C21—C20—C25	117.4 (3)
C5—C6—H6A	108.8	C21—C20—C17	123.5 (3)
C1—C6—H6A	108.8	C25—C20—C17	119.1 (3)
C5—C6—H6B	108.8	C22—C21—C20	121.6 (5)
C1—C6—H6B	108.8	C22—C21—H21A	119.2
H6A—C6—H6B	107.7	C20—C21—H21A	119.2
C12—C7—C8	118.0 (4)	C23—C22—C21	121.0 (5)
C12—C7—C5	118.8 (3)	C23—C22—H22A	119.5
C8—C7—C5	123.2 (4)	C21—C22—H22A	119.5
C7—C8—C9	120.4 (5)	C22—C23—C24	118.8 (4)
C7—C8—H8A	119.8	C22—C23—H23A	120.6
C9—C8—H8A	119.8	C24—C23—H23A	120.6
C10—C9—C8	120.8 (5)	C23—C24—C25	120.7 (5)
C10—C9—H9A	119.6	C23—C24—H24A	119.7
C8—C9—H9A	119.6	C25—C24—H24A	119.7
C9—C10—C11	119.4 (5)	C20—C25—C24	120.4 (5)
C9—C10—H10A	120.3	C20—C25—H25A	119.8
C11—C10—H10A	120.3	C24—C25—H25A	119.8
C10—C11—C12	120.1 (6)	C31—C26—C27	118.3 (3)
C10—C11—H11A	120.0	C31—C26—C13	120.5 (3)
C12—C11—H11A	120.0	C27—C26—C13	121.2 (3)
C7—C12—C11	121.4 (5)	C28—C27—C26	121.0 (3)
C7—C12—H12A	119.3	C28—C27—H27A	119.5
C11—C12—H12A	119.3	C26—C27—H27A	119.5
C26—C13—C2	112.5 (2)	C29—C28—C27	120.9 (3)
C26—C13—C14	110.6 (2)	C29—C28—H28A	119.6
C2—C13—C14	109.4 (2)	C27—C28—H28A	119.6
C26—C13—H13A	108.1	C30—C29—C28	117.9 (3)
C2—C13—H13A	108.1	C30—C29—H29A	121.1
C14—C13—H13A	108.1	C28—C29—H29A	121.1
C19—C14—C15	107.6 (2)	C29—C30—C31	123.0 (3)
C19—C14—C13	111.5 (2)	C29—C30—N1	119.0 (3)
C15—C14—C13	114.2 (2)	C31—C30—N1	117.9 (3)
C19—C14—H14A	107.8	C30—C31—C26	118.9 (3)
C15—C14—H14A	107.8	C30—C31—H31A	120.5
C13—C14—H14A	107.8	C26—C31—H31A	120.5
O2—C15—C16	123.8 (3)	O6—N1—O5	123.1 (4)
O2—C15—C14	121.9 (3)	O6—N1—C30	118.5 (3)
C16—C15—C14	114.3 (3)	O5—N1—C30	118.4 (4)
C15—C16—C17	111.7 (3)	C3—O3—C19	115.4 (2)
C15—C16—H16A	109.3	C19—O4—H4C	109.5
C17—C16—H16A	109.3		
			
O1—C1—C2—C3	172.3 (3)	C16—C17—C18—C19	−55.4 (3)
C6—C1—C2—C3	−0.5 (4)	C17—C18—C19—O4	−61.8 (3)
O1—C1—C2—C13	−2.1 (4)	C17—C18—C19—O3	178.2 (2)
C6—C1—C2—C13	−175.0 (3)	C17—C18—C19—C14	59.2 (3)
C1—C2—C3—O3	−168.0 (2)	C15—C14—C19—O4	68.2 (3)
C13—C2—C3—O3	6.1 (4)	C13—C14—C19—O4	−57.8 (3)
C1—C2—C3—C4	11.1 (4)	C15—C14—C19—O3	−173.4 (2)
C13—C2—C3—C4	−174.8 (3)	C13—C14—C19—O3	60.6 (3)
C2—C3—C4—C5	10.0 (4)	C15—C14—C19—C18	−56.7 (3)
O3—C3—C4—C5	−170.8 (3)	C13—C14—C19—C18	177.3 (2)
C3—C4—C5—C6	−40.7 (4)	C16—C17—C20—C21	−38.2 (5)
C3—C4—C5—C7	−172.6 (3)	C18—C17—C20—C21	86.3 (5)
C4—C5—C6—C1	51.0 (5)	C16—C17—C20—C25	143.3 (3)
C7—C5—C6—C1	−178.6 (3)	C18—C17—C20—C25	−92.1 (4)
O1—C1—C6—C5	155.9 (4)	C25—C20—C21—C22	1.8 (8)
C2—C1—C6—C5	−31.1 (5)	C17—C20—C21—C22	−176.7 (4)
C6—C5—C7—C12	161.1 (4)	C20—C21—C22—C23	−0.1 (9)
C4—C5—C7—C12	−68.7 (5)	C21—C22—C23—C24	−0.7 (9)
C6—C5—C7—C8	−16.1 (6)	C22—C23—C24—C25	−0.2 (8)
C4—C5—C7—C8	114.1 (4)	C21—C20—C25—C24	−2.7 (6)
C12—C7—C8—C9	−0.9 (6)	C17—C20—C25—C24	175.9 (4)
C5—C7—C8—C9	176.3 (4)	C23—C24—C25—C20	2.0 (7)
C7—C8—C9—C10	0.1 (6)	C2—C13—C26—C31	−51.4 (3)
C8—C9—C10—C11	0.4 (7)	C14—C13—C26—C31	71.3 (3)
C9—C10—C11—C12	−0.2 (8)	C2—C13—C26—C27	131.3 (3)
C8—C7—C12—C11	1.2 (7)	C14—C13—C26—C27	−106.0 (3)
C5—C7—C12—C11	−176.2 (4)	C31—C26—C27—C28	−0.4 (5)
C10—C11—C12—C7	−0.7 (8)	C13—C26—C27—C28	176.9 (3)
C3—C2—C13—C26	128.5 (3)	C26—C27—C28—C29	0.8 (5)
C1—C2—C13—C26	−57.3 (3)	C27—C28—C29—C30	−0.5 (5)
C3—C2—C13—C14	5.2 (4)	C28—C29—C30—C31	−0.2 (5)
C1—C2—C13—C14	179.3 (2)	C28—C29—C30—N1	−179.3 (3)
C26—C13—C14—C19	−162.0 (2)	C29—C30—C31—C26	0.6 (5)
C2—C13—C14—C19	−37.5 (3)	N1—C30—C31—C26	179.7 (3)
C26—C13—C14—C15	75.8 (3)	C27—C26—C31—C30	−0.2 (4)
C2—C13—C14—C15	−159.8 (2)	C13—C26—C31—C30	−177.5 (3)
C19—C14—C15—O2	−123.8 (3)	C29—C30—N1—O6	161.0 (4)
C13—C14—C15—O2	0.6 (4)	C31—C30—N1—O6	−18.2 (5)
C19—C14—C15—C16	54.7 (3)	C29—C30—N1—O5	−18.4 (5)
C13—C14—C15—C16	179.0 (2)	C31—C30—N1—O5	162.4 (3)
O2—C15—C16—C17	123.6 (3)	C2—C3—O3—C19	17.6 (4)
C14—C15—C16—C17	−54.8 (4)	C4—C3—O3—C19	−161.6 (2)
C15—C16—C17—C20	178.2 (3)	O4—C19—O3—C3	66.2 (3)
C15—C16—C17—C18	53.3 (4)	C18—C19—O3—C3	−171.5 (2)
C20—C17—C18—C19	177.8 (3)	C14—C19—O3—C3	−50.2 (3)

### Hydrogen-bond geometry (Å, º)

**Table:**

*D*—H···*A*	*D*—H	H···*A*	*D*···*A*	*D*—H···*A*
O4—H4*C*···O1^i^	0.82	1.89	2.714 (3)	180

Symmetry code: (i) *x*−1/2, −*y*+1/2, −*z*.
